# Elucidating the Role of SET as a Key Contributor to Neurodevelopmental Disability Within the 9q34.11 Deletion Syndrome Interval

**DOI:** 10.1111/cge.70213

**Published:** 2026-07-10

**Authors:** Angelo Condell, Elaine Zhang, Tim Sikora, Sean Massey, Nicole J. Van Bergen, Min Wang, Cas Simons, Katrina M. Bell, Daniella H. Hock, David A. Stroud, David Francis, Wendy A. Gold, Martin B. Delatycki, John Christodoulou, Simranpreet Kaur

**Affiliations:** ^1^ Brain and Mitochondrial Research Group Murdoch Children's Research Institute Melbourne Victoria Australia; ^2^ Canberra Clinical Genomics The Australian National University, Canberra Health Services Canberra Australian Capital Territory Australia; ^3^ Murdoch Children's Research Institute, Royal Children's Hospital Melbourne Victoria Australia; ^4^ Department of Paediatrics University of Melbourne Melbourne Victoria Australia; ^5^ Centre for Population Genomics Garvan Institute of Medical Research and UNSW Sydney Sydney New South Wales Australia; ^6^ Bio21 Molecular Science and Biotechnology Institute University of Melbourne Melbourne Victoria Australia; ^7^ Victorian Clinical Genetics Service Royal Children's Hospital Melbourne Victoria Australia; ^8^ School of Medical Sciences and Discipline of Child and Adolescent Health, Faculty of Medicine and Health The University of Sydney New South Wales Australia; ^9^ Molecular Neurobiology Research Laboratory, Kids Research The Children's Hospital at Westmead, and The Children's Medical Research Institute New South Wales Australia; ^10^ Kids Neuroscience Centre, Kids Research The Children's Hospital at Westmead New South Wales Australia

**Keywords:** 9q34.11 deletion, intellectual disability (ID), neurodevelopmental disorders (NDDs), Rett syndrome (RTT), *SET*, *SET* proto‐oncogene, speech therapy

## Abstract

The 9q34.11 chromosomal region contains multiple neurodevelopmental genes involved in synaptic transmission, axonal structure and neuronal maturation. Pathogenic microdeletions, duplications and single nucleotide variants in numerous genes were previously linked with neurodevelopmental disorders (NDDs). Amongst them, *SET* has recently been implicated in a rare NDD with speech delay and facial dysmorphism. This study reports a female with a heterozygous *de novo* deletion impacting *SET* but not other NDD‐associated genes at 9q34.11. The proband was initially diagnosed with atypical Rett syndrome with overlapping clinical features of *SET* haploinsufficiency. The deletion was confirmed using microarray and long‐read sequencing. Subsequent quantitative proteomic evaluation identified a significant decrease of SET protein in patient‐derived fibroblasts compared to control lines. This study provides insights into the proband's clinical course over their 28 year diagnostic odyssey, and emphasises the benefits of early speech therapy interventions. The proband had no functional speech, but regained the capacity to meaningfully communicate and articulate a limited vocabulary in adulthood, concordant with other reported non‐paediatric cases of *SET*‐NDD. This study expands current knowledge on the genotypic and phenotypic spectra of *SET*‐NDD, and pinpoints a smaller 9q34.11 critical region excluding upstream NDD‐associated genes, *STXBP1* and *SPTAN1*, implicating *SET* as a significant NDD‐associated gene.

## Graphical abstract

This study reports a female proband with a *de novo* 9q34.11 deletion affecting *SET*, who underwent a 28‐year diagnostic odyssey after an atypical Rett syndrome clinical diagnosis. Genomic and proteomics analyses confirmed *SET* haploinsufficiency, refining the critical 9q34.11 region, and supporting speech therapy benefits in improving meaningful communication in adulthood.

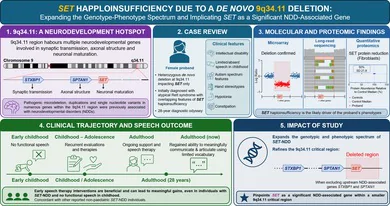

## 1 Introduction

Intellectual disability (ID) affects approximately 1%–2% of the global population, with prevalence rates varying between different environments, demographics, and socioeconomic contexts [1, 2]. In Australia, current local NDD prevalence estimates span 0.85%–1.7% [3, 4]. These overarching statistics encapsulate a broad range of clinically diverse and phenotypically heterogeneous NDDs, some of which may overlap in symptomatology, potentially leading to a lengthy diagnostic odyssey for patients and families, with challenges in pinpointing conclusive diagnoses and predicting disease course [5].

To address this, next generation sequencing (NGS) techniques are helping to increase the diagnostic yield of NDDs, leading to a rapid expansion of genomic knowledge, whilst proving beneficial for informative, timely and family‐centric diagnostic genetic results [2, 5, 6]. Additionally, as costs decrease and technology improves over time, long‐read sequencing (LRS) may also be an emerging option in the genomic investigation of NDDs, with the added benefit of copy‐number (CNV) and structural (SV) variant detection [7, 8, 9].

A notable area of rapid progress in identifying the mechanistic pathogenesis of NDDs relates to genes involved in the dynamic processes of chromatin remodelling, and the co‐packing of DNA with histones into compact nucleosome superstructures [10, 11]. Nucleosome assembly proteins (NAPs) are one such example, influencing neuronal function and differentiation through their involvement in histone and nucleosome modification, chromatin structure, epigenetic regulation of transcriptional activation and/or repression, and other interactions within brain‐specific developmental pathways [12, 13, 14], making them potential candidates for NDD disease‐gene associations. Of particular interest is the ‘*SET*’ nuclear proto‐oncogene (*SET*), a key chromatin remodelling factor originally characterised in genomic translocations of acute myeloid leukaemia, and now recognised to be functionally critical for DNA methylation and transcriptional silencing as a component of the inhibitor of histone acetyltransferases (INHAT) complex [10, 15, 16]. As a NAP, SET also acts as a chaperone to prevent the unfolding or aberrant aggregation of histones, and is also critical in the growth, maturation and differentiation of neurons [12, 14]. Recent reports of rare and novel loss‐of‐function (LoF) genetic variation in *SET*—predominantly small insertion‐or‐deletion (indel) frameshift variants, as well as nonsense, splice‐affecting, and missense single nucleotide variants (SNVs)—have solidified this gene as a relatively‐new aetiology of autosomal dominant ID/NDD, with at least 50% of the diagnosed cases harbouring *de novo* variants [10, 16, 17, 18].

Currently, the DECIPHER database [19] lists multiple individuals affected by NDD with large structural variants (SVs) also containing *SET*. However, narrowing down the critical NDD region in the 9q34.11 region has been a challenge. Larger copy‐number variants (CNVs) upstream of *SET* impact other NDD‐associated genes, such as Syntaxin‐Binding Protein 1 (*STXBP1*) [20]. Deletions at 9q34.11 that do *not* impact *STXBP1* are relatively rare. There has been at least one recent case report documenting a large 2.0 Mb *de novo* deletion at 9q34.11, which impacts *SET* and an upstream NDD‐associated gene Spectrin Alpha, Non‐Erythrocytic 1 (*SPTAN1*), but not *STXBP1* [21], suggesting these other genes may also be contributing to the clinical variability of NDD phenotypes associated with deletions involving the 9q34.11 region.

This study identified a small NDD critical region surrounding *SET* using microarray, singleton whole genome sequencing (WGS) and Oxford Nanopore LRS. We describe for the first time a proband with a 326 kb *de novo* deletion at 9q34.11, which deletes *SET* completely but does not impact the NDD‐associated genes *STXBP1* or *SPTAN1*, leading to a clinical picture reminiscent of atypical Rett syndrome (RTT) and the recently‐documented *SET*‐associated NDD [10, 22]. This study also provides functional validation of *SET* protein reduction through mass spectrometry‐based proteomics analysis using patient‐derived fibroblasts, and provides long‐term clinical observations into the phenotypic course of this disorder through the 28‐year clinical progression of the female proband.

## 2 Materials and Methods

### 2.1 Recruitment and Consent

Methods of this study were approved by the Human Research Ethics Committee of the contributing institutes in accordance with the Helsinki Declaration of 1975, as revised in 2000. The proband was recruited at Victorian Clinical Genetic Services (VCGS; VIC, Australia) by Prof Martin B. Delatycki. Clinical phenotypes were collected by Prof Martin B. Delatycki from the proband's initial presentation to the time of manuscript publication. Written informed consent for participation in the study and for the presentation and publication of clinical information was obtained from the proband's legal guardian.

### 2.2 Initial SNP Microarray and Exome Sequencing

Trio molecular karyotyping was performed in 2018 using gDNA extracted from patient blood by the NATA‐accredited Cytogenetics Laboratory at the VCGS. Whole genome SNP microarray was performed using the Illumina Infinium HumanCoreExome‐12 BeadChip v1.1 platform (approximately 650 000 SNP markers) and was analysed using KaryoStudio software version 1.4 [genome assembly GRCh37/hg19]. Singleton whole exome sequencing (WES) was performed as previously reported [23].

### 2.3 Singleton Whole Genome Sequencing (WGS)

Singleton whole genome sequencing (WGS) was performed in 2025 using gDNA extracted from patient fibroblasts at VCGS. SNVs/indels were called using v4.2 GATK best practice pipeline (genome build GRCh38) and was uploaded to an open‐sourced web‐based variant curation platform, *seqr*, for analysis [24]. Short tandem repeats (STRs), mitochondrial variants, and SVs analyses were conducted using the v2.2 STRipy [25], GATK MuTect2‐generated MitoReport [26, 27], and Ximmer/CXGo platforms [28], respectively. Variant prioritisation and curation involved the selection and evaluation of variants from specific gene panels (Table S1.1) [29], based on their population frequency, zygosity, *in silico* predictions scores, gene constraints, regional conservations, functional validations, and ClinVar classifications [24, 30, 31, 32, 33]. Mismatched soft‐clipped sequences were re‐aligned using BLAT and the UCSC Genome Browser [32], and manual examination of reads spanning the deletion area was conducted via IGV [34].

### 2.4 Nanopore Long‐Read Sequencing (LRS)

Long‐read sequencing (LRS) was performed on gDNA extracted from patient fibroblasts using Oxford Nanopore ‘Read Until’ selective sequencing [35]; such adaptive sampling allowed for the generation of long genomic sequence reads spanning the estimated breakpoints of the previously‐identified deletion. Two target regions were examined at either end of the 9q34.11 region of interest, one encompassing the *SET* gene ± ≈40 kb (GRCh38: chr9:128643663‐128743663) close to the 5′ end of the deletion, and the other encompassing the *NUP188* gene ± ≈50 kb (GRCh38: chr9:128897699‐129057096) at the 3′ end, so that multi‐kb reads spanning the region of interest could be covered at a reasonable cost. Phasing was performed by WhatsHap [36]. SNVs/indels and SVs were called using Clair3 and NanoSV respectively [36, 37]. SVs were bioinformatically filtered out using R if the position was outside the Nanopore capture regions, or if the SV was common in gnomAD v4 or in this segregation cohort [30]. Mismatched soft‐clipped sequences were re‐aligned using BLAT and the UCSC genome browser [32, 38], and manual examination of reads spanning the deletion area was conducted via IGV [34].

### 2.5 Fibroblast Tissue Culture and Cell Harvesting

Primary cultures of fibroblasts from proband and unrelated controls were established from skin biopsies as previously described [39]. Patient‐derived skin fibroblasts were cultured in Dulbecco's Modified Eagle Medium (DMEM; Sigma #D6546), with 10% v/v foetal bovine serum (FBS; Gibco #10099141), 3.7 g/L NaHCO_3_, 100 units/mL penicillin and 100 μg/mL streptomycin. Cultures were maintained in a humidified incubator at 37°C and 5% CO_2_, and harvested by trypsinisation (0.025% trypsin/EDTA) at ~90% confluency, centrifuged at 500*g* for 5 min. Cells were pelleted at 500*g* for 5 min at 4°C, washed with PBS and snap‐frozen at −80°C for further proteomic investigations.

### 2.6 Mass Spectrometry‐Based Proteomics Analysis

Mass spectrometry proteomic analysis was performed using the patient fibroblasts sample in triplicate and five unrelated controls as previously described [40]. Briefly, fibroblasts pellets were lysed and proteins digested using S‐Trap micro columns (Protifi LLC) according to manufacturer's instructions. Briefly, cell pellets were solubilised in 5% (w/v) SDS, 50 mM TEAB (pH 8.5). Proteins were reduced and alkylated using 10 mM tris(2‐carboxyethyl)phosphine (TCEP) and 40 mM 2‐chloroacetamide (CAA) and incubated at 37°C with trypsin at 1:25 trypsin to protein ratio. Raw mass spectrometry data was acquired by liquid chromatography (LC)‐tandem mass spectrometry (MS/MS) on an Orbitrap Eclipse Mass Spectrometer (Thermo Fisher Scientific) using a previously described LC setup for data‐independent analysis (DIA) [40]. Raw data was processed using Spectronaut software version 20.1.250624.92449 (Kuiper) (Biognosys) with default directDIA settings with exception of ‘Protein Qvalue Cutoff (Run)’ set to 0.01, ‘Major and Minor Group Top N’ was unselected and ‘directDIA workflow’ set to directDIA+ (deep). Peptide abundance plot was generated in RStudio using ggplot package as previously [41]. Protein abundance plot was generated in RStudio as previously using canonical proteins [40].

## 3 Results

### 3.1 Clinical Summary

Table 1 summarises key clinical details of the proband compared to others reported with SNVs leading to *SET*‐associated NDD (at least 15 other patients at the time of publication) [10, 16, 17, 18, 21, 42, 43], alongside a key CNV case report of a proband with a deletion in the 9q34.11 region impacting *SET* and *SPTAN1* but not *STXBP1* [21], and a DECIPHER patient with a partial deletion of *SET* only (Patient #265418) [19]. Additional phenotypic information can be found in Tables S2.1 and S2.2.

**TABLE 1:** Clinical features and genomic details of previously reported neurodevelopmental disorder patients harbouring *SET* variants.

Variant type	Study	Patient number	Genetic variant (SET)	Developmental delay	Intellectual disability	Language deficits	Motor deficits	Behavioural disorders	Epilepsy	Facial abnormalities
SNVs, indels	Hamdan et al. (2014) [17]	1	c.699_701del; p.(Tyr233Ter)	+	+	+	+	+	−	+
	Stevens et al. (2018) [10]	1A	c.167_170del; p.(Arg57LeufsTer10)	+	+	+	+	−	−	+
		1B *(Mother of 1A)*		N/A	+	+	N/A	−	−	+
		2	c.283T>G; p.(Trp95Gly)	+	+	+	+	−	−	+
		3	c.352C>T; p.(His118Tyr)	+	+	+	+	+	−	+
		4	c.417+1G>C	+	+	+	+	−	−	+
		5	c.689_690dup; p.(Gln231TyrfsTer29)	+	+	+	+	−	+	+
	Richardson et al. (2018) [16]	1	c.167_170del; p.(Arg57LeufsTer10)	+	+	+	+	−	−	+
		2	c.167_170del; p.(Arg57LeufsTer10)	+	+	+	+	+	−	+
		3	c.459_460del; p.(Lys154ArgfsTer6)	+	+	+	+	+	−	+
	Marinakis et al. (2021) [42]	9151	c.99_102del; p.(Arg35LeufsTer10)	+	+	+	+	N/A	N/A	+
	Pan et al. (2023) [18]	1	c.532‐3T>A	N/A	+	+	N/A	N/A	−	+
		2	c.3G>C; p.0?	N/A	+	N/A	N/A	N/A	−	+
Deletion CNVs	DECIPHER [19]	265 418	chr9:128690919‐128694723del	N/A	+	N/A	N/A	N/A	N/A	+
	Shono et al. (2022) [21]	1	chr9:128222769‐130233381del	+	+	+	+	−	−	+
	This study	1	chr9:128656218‐128981804del	+	+	+	+	+	−	+

*Note:* All variants listed are heterozygous. All variants listed are also de novo except 1A (inherited from the affected de novo mother 1B), both Pan et al. cases (parental data unavailable) [18], and the DECIPHER case 265 418 (unknown inheritance) [19]. Coordinates from the Shono et al. case have been converted into GRCh38 from GRCh37 [21]. McRae et al. did not provide detailed patient information and is not described in the table and it is unclear whether these patients are those previously reported [43]. Standard deviation (SD) is unavailable for height, weight and OFD in the Pan et al. cases [18], nor for our proband. Transcript used for *SET* SNVs (c. shown in table) is NM_001122821.2, the longest clinically‐relevant transcript encoding NP_001116293.1 (isoform 1, p. shown) (as opposed to the MANE transcript NM_003011.4 encoding isoform 2), as this is used by all other previously published case reports. CNV coordinates are provided in GRCh38/hg38, with approximate deletion size also noted. Features are confirmed to be present using ‘+’ and absent using ‘−’ unless (unless otherwise stated to be ‘implied’), N/A indicates no data/unknown/unsure if present or absent due to insufficient details. Further details available in Table S2.2. Additional phenotypic information can be retrieved by directly accessing the relevant publications. In Table S2.2: *Heights and weights for patients in Richardson et al. were measured at younger ages: 5 years for Patient 1, 16 years for Patient 2, and 9 years for Patient 3—the same ages are used for OFD except for **Patient 2, whose OFD was measured only at 5 years; and, the paper only provided percentiles without any raw data on height or weight [16].

Abbreviations: ADD: Attention Deficit Disorder (without hyperactivity); CNVs: copy number variants, where SET is the main (or only, in the DECIPHER case) candidate gene implicated within the region; EEG: electroencephalogram; F: female; indels: small insertion/deletion variants; LOC: loss‐of consciousness events; M: male; OFD: occipito‐frontal diameter; RTT‐like: Rett syndrome like feature; SNVs: single nucleotide variants.

#### 3.1.1 Initial Presentation

This female proband presented in infancy with an atypical Rett syndrome (RTT) NDD phenotype (Table S3.1). She was late to ‘sit and roll’ (≈4.5 months of age) and displayed global developmental delay (≈7 months of age). Gross and fine motor skills regressed, requiring physiotherapy intervention. There was no functional speech initially, which showed some development with intensive speech therapy to allow articulation of simple words. With time, it became apparent that she had severe ID associated with laughing/screaming spells, with an additional diagnosis of autism spectrum disorder (ASD) being made. She developed other RTT features, including hand stereotypies, gait deterioration, sleep disturbances, bruxism, peripheral vasomotor disturbances, small cold hands and feet, episodic hyperventilation, diminished pain response, and intense eye‐pointing. Brain Magnetic Resonance Imaging (MRI) did not show any significant anomalies, and the proband has no history of seizures or congenital organ defects. Additional clinical details can be found in Supporting Information S3.

#### 3.1.2 Long‐Term Outcomes

Contact with the proband and her clinical team was recently re‐established at the age of 28. Although the proband still displays severe ID, her condition has remained stable and consistent for several years, with minimal phenotypic change. Regarding motor skills, the proband is ambulant, but fatigues easily and can therefore only walk short distances at a time: she usually cannot walk more than 200 m at once, but on a good day will be able to achieve up to 2000 steps. Additionally, hand function has now declined in recent years, and she now finds it difficult to hold and drink from a cup, and generally hand‐feeds rather than using cutlery, which she could achieve at an earlier age. She has very limited speech, but she can still communicate some phrases (e.g., ‘in the car’, ‘go away’) and use several noises that her family can interpret with meaning. She currently has significant constipation and is on multiple medications to treat this. Additional previously undescribed features include kyphosis and hypotonia with slight growth deficiency without joint laxity. The proband has not had any seizures (normal electroencephalography), although she has experienced two episodes of unexplained loss‐of‐consciousness. Accompanying her ASD, she now presents with significant anxiety, which is managed with medications (lyrica, risperidone, frisium) with positive results. She now lives in supported accommodation and is reported to be happy and settled in this environment.

### 3.2 Facial Phenotype

**FIGURE 1:**
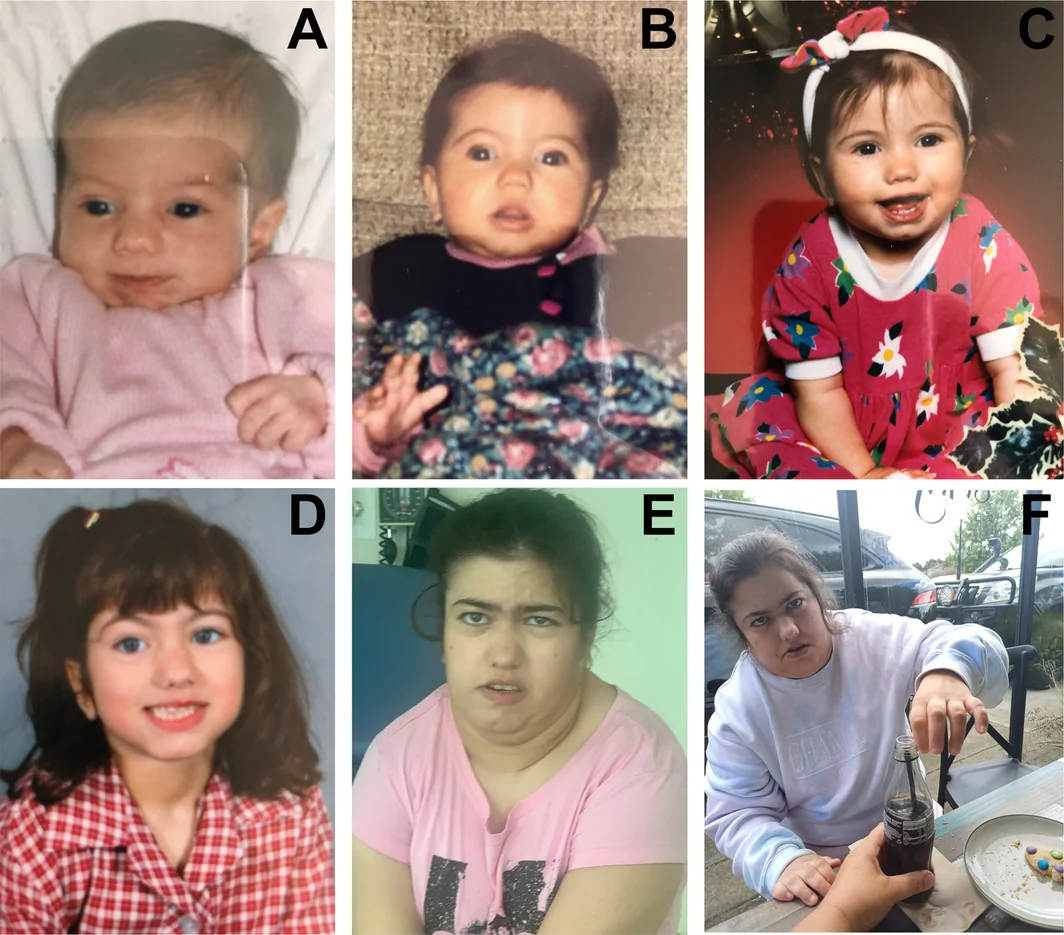
Photos of the proband from infancy to adulthood. (A–F) Notable features are a broad nasal tip, broad columella, synophrys and thick medial eyebrows.

The proband displays facial asymmetry and has had a significant squint (strabismus) since the age of 2 years (Figure 1). Other features include broad nasal tip, synophrys, a dimple in the chin, and broad columella. The proband does not show significant microcephaly (head circumference 75th percentile), nor any clefting or palatal anomalies.

### 3.3 Genomic Findings

Previous trio WES and singleton WGS did not identify any SNVs or indels impacting the known RTT‐associated genes Methyl‐CpG binding protein 2 (*MECP2*), Cyclin Dependent Kinase‐like 5 (*CDKL5*), or Forkhead Box G1 (*FOXG1*) in this proband [22]. Singleton WGS was conducted in the proband only to ensure that no additional plausible explanations for the proband's phenotypes were overlooked. Both singleton WGS and microarray data confirmed no CNVs present in these genes, indicating a genetically negative status for current genes associated with RTT or atypical RTT syndrome in this patient.

### 3.4 Deletion at 9q34.11 Encompasses the SET Proto‐Oncogene

**FIGURE 2:**
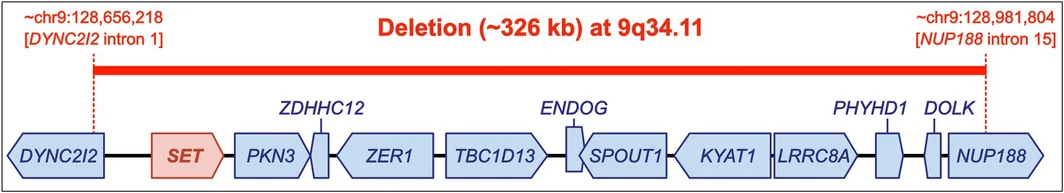
Assessment of key RefSeq‐named genes located within the proband's heterozygous de novo ≈326 kb deletion at 9q34.11, with the NDD candidate gene SET shown in red. Breakpoints provided are in GRCh38/hg38. Arrow‐ends of each gene depicted indicate the directionality of that gene in the genome (forward or reverse strand). This diagram does not include uncharacterised long non‐coding RNAs (lncRNAs) which may be present in this region (e.g., LOC100506100) (see Figure 3).

**FIGURE 3:**
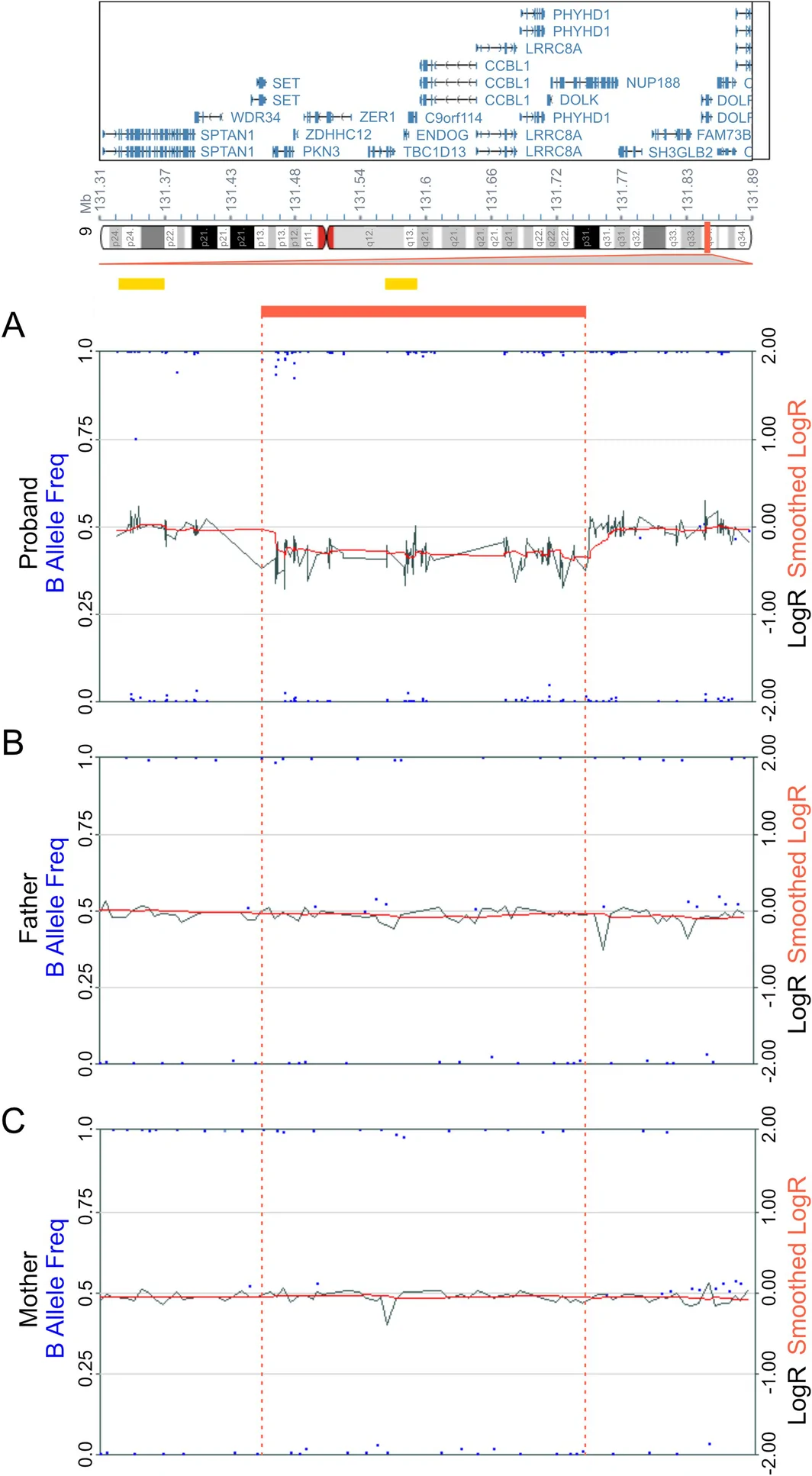
Microarray profile of the 9q34.11 deletion (orange rectangle on chromosome view) shown in Illumina KaryoStudio. Local genes and other genomic features (including long non‐coding RNAs) are listed in blue in the top panel. The deleted region boundaries are depicted by vertical serrated orange lines. Blue dots indicate the B allele frequency. Horizontal orange lines indicate the smoothed logR tracking along deleted region compared to the black lines which indicate the raw logR, shown in the proband (A) compared with the paternal (B) and maternal (C) samples. Annotations are added through Affinity Designer 2 (v.2.6.5; https://www.affinity.studio/graphic‐design‐software).

An initial SNP microarray and subsequent Nanopore LRS confirmation identified a *de novo* heterozygous ≈326 kb deletion at cytoband 9q34.11 [GRCh38/hg38: approx. chr9:128656218‐128981804], with breakpoints spanning from Dynein 2 Intermediate Chain 2 (*DYNC2I2*) to Nucleoporin 188 (*NUP188*), impacting 13 genes in total (Figures 2 and 3; Table S3). Each gene in the 9q34.11 deletion interval was analysed in detail for phenotypic and molecular/functional relevance and the potential for LoF‐based pathogenicity (Table S3). Of these genes, the most clinically significant NDD‐associated candidate gene was *SET*, a highly conserved gene playing a critical role in apoptosis, transcription, and nucleosome assembly (Table S3; Figure S3.1), which is recently associated with an autosomal dominant intellectual developmental disorder in OMIM (#600960) and PanelAPP (listed as Green, or high‐confidence, diagnostic‐grade for ID in both the Genomics England and Australian releases) [29, 44]. Notably, this deletion does not include the other previously reported 9q34.11 NDD critical region genes upstream of *SET*, such as *STXBP1* or *SPTAN1*.

Initially, another NDD‐associated gene in the interval, *NUP188*, was proposed as an additional candidate gene due to the presence of a paternally inherited missense variant. This variant was later found to be *in cis* with the *de novo* deletion through LRS. In addition, Western blot and proteomics analysis were supportive of decreased protein levels; however, immunofluorescent studies were not supportive of decreased NUP188 protein (see Supporting Information S4 for further discussion).

Using the Riggs et al. ClinGen guidelines for the diagnostic classification of constitutional CNVs [45], this deletion was classified as *Pathogenic* (proband copy loss score 1.15 > 0.99 pathogenicity threshold) by applying criteria 1A (copy loss containing protein coding genes; +0.00); 2A (complete overlap of at least one established haploinsufficient (HI) gene *SET* [10, 16, 17, 18, 21]; +1.00), 3A (deletion contains 13 genes, falling within the 0–24 interval; +0.00), and 5A (confirmed *de novo*, phenotype is consistent but not specific with the *SET‐associated NDD*; +0.15).

### 3.5 Proteomics Analysis Identifies Haploinsufficiency of Proteins in the Deletion Region

**FIGURE 4:**
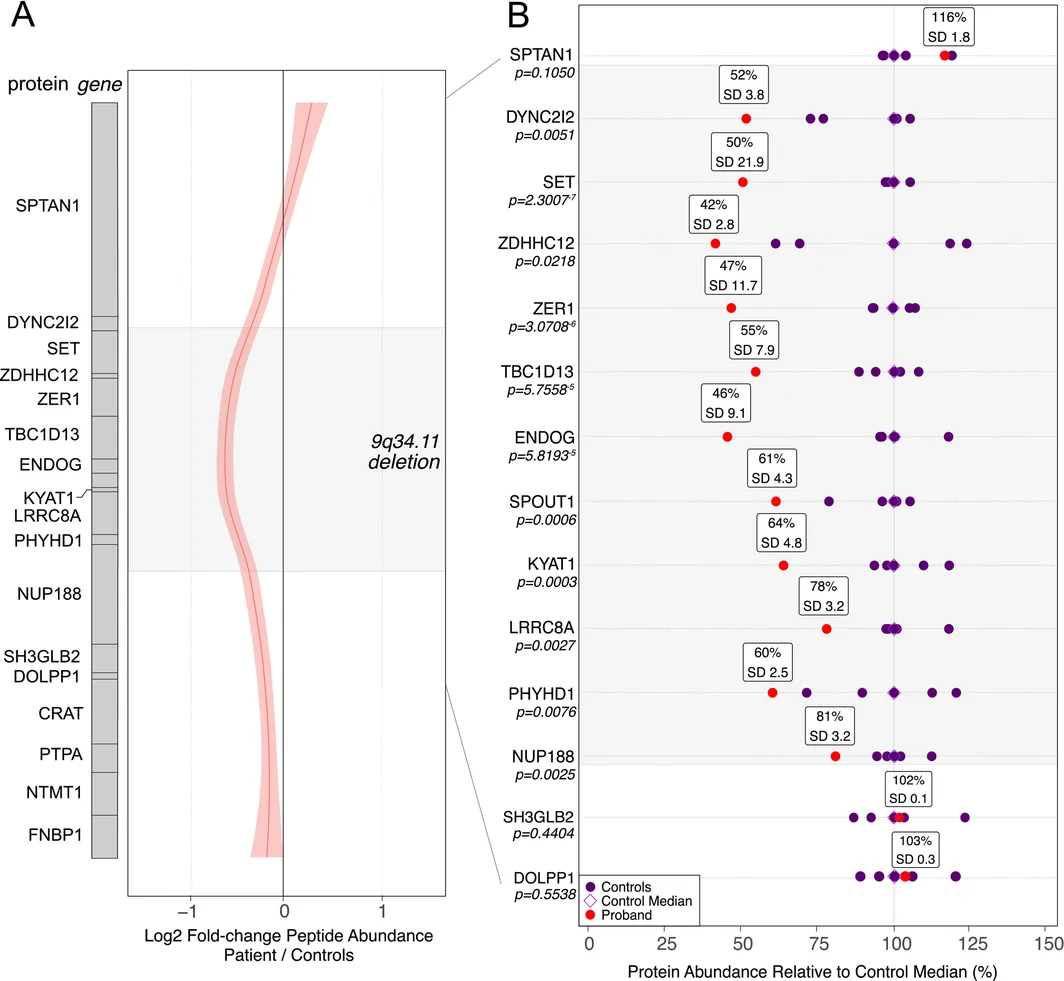
(A) Log2 fold‐change of the peptide abundances in patient fibroblasts compared to controls (*n* = 5) plotted using loess method [41] and 95% confidence interval. Grey shaded area indicates peptides encoded by genes present in the proband's heterozygous 9q34.11 deletion interval and flanking genes. (B) Abundance range of proteins located within the 9q34.11 deletion interval showing reduction in the proteins within the deletion while proteins from the flanking genes SPTAN1, SH3GLB2 and DOLPP1 were unchanged. *p*‐value calculated from a two‐sample two‐tailed *t*‐test. SD = standard deviation.

Log_2_ fold‐change of the peptides representing proteins mapped to the 9q34.11 deletion region and flanking genes demonstrate a reduction in abundance of peptides in proband relative to controls (*n* = 5) within the deletion region (Figure 4A). At the protein level, a reduction in abundance is also seen across all proteins encoded by genes within the 9q34.11 deletion (range 46%–81% and outside control range) while proteins encoded by flanking genes (*SPTAN, SH3GLB3, DOLPP1*) are within control range (Figure 4B). Whole‐cell levels of the canonical SET protein are reduced to 50% and present the highest standard deviation (SD 21.9) out of all proteins within the deletion region in proband fibroblasts relative to controls.

## 4 Discussion

### 4.1 Establishing *SET* as the Critical Gene at 9q34.11

This study describes the first proband with atypical RTT features who carries a heterozygous *de novo* deletion at 9q34.11, which includes the *SET* gene but not other previously documented NDD associated genes such as *STXBP1* and *SPTAN1* that have been implicated in larger deletion intervals [46, 47]. Proteomic investigation also demonstrated a statistically significant decrease in the level of SET protein in proband fibroblasts compared to control lines, while SPTAN1 protein, which is outside of the deletion region, was unchanged. This further strengthens the involvement of *SET* as a critical gene in the overarching 9q34.11 region for NDD phenotypes.

### 4.2 Comparison With Other *SET* ‐NDD Cases

There is considerable phenotypic overlap between the proband and previous cases of *SET*‐NDD in the literature. Core features shared across cases include difficulties with speech and ambulation, ID and developmental delay without seizures, and key facial dysmorphic features such as facial asymmetry and strabismus (Table 1) [10, 16, 17, 18, 42, 43]. Hypotonia also appears to be emerging as a recurring feature of the condition [10, 16, 17, 18, 42, 43] [DECIPHER #265418].

Attention should be drawn to the varying nature of the speech difficulties reported across the developmental stages of multiple cases. Severe speech delay or speech inability during early years seems to be a core feature of *SET‐*NDD [16, 17, 18]. However, some patients have retained or even regained the ability to speak short sentences with meaningful vocabulary, including a repertoire from two [21] to 20 words [17]. Most notable is Patient 1b reported in Stevens et al., the 49 year old mother of a similarly affected child, who overcame speech delay from childhood to the ‘good speech ability’ in present with the capacity to read short basic segments of text [10]. Similarly, our proband, who had no functional speech in early years, has recovered the ability to articulate numerous words with early intervention of intensive speech therapy, and can now use several meaningful phrases (as well as noises). These long‐term insights both in the proband and others in the literature would suggest that language skills may gradually improve in *SET*‐NDD probands over time if speech therapy is pursued despite initial delays and general limitations, and might prove beneficial for patients as a treatment option as they progress [16, 17, 18]. Further studies are required to discern which forms of speech therapy hold the most potential to assist communicative ability in those affected by *SET*‐NDD.

However, our proband does display some differences to other *SET*‐NDD cases. This proband consistently displayed a clinical profile indicative of atypical RTT from an early age; RTT has not been explicitly noted as an alternative diagnosis in other *SET*‐NDD reports thus far. Additionally, our proband meets several minor diagnostic criteria of RTT (e.g., bruxism, small cold hands and feet, diminished pain responses), which have not been reported in other *SET*‐NDD cases. Conversely, some recurring *SET‐*NDD features are not reported in this proband (e.g., digit anomalies, joint laxity), acknowledging though that *SET‐*NDD is a phenotype with a high degree of clinical variability [10, 16, 17, 18, 42, 43].

### 4.3 Comparison With Other 9q34.11 Deletion Reports

Aside from the overlap with *SET*‐NDD, this proband also shares clinical features with other affected individuals harbouring deletions at the 9q34.11 cytoband. Whilst there are phenotypic similarities, these deletions can span different genes at 9q34.11, including other NDD‐associated genes such as *SPTAN1* and *STXBP1* [20, 21, 48]. However, as postulated by Ehret et al. [20], *STXBP1* is not the only causative gene at the 9q34.11 locus, and deletions of varying sizes not including *STXBP1* or *SPTAN1* may also be relevant for NDD phenotypes, such as those affecting *SET*.

There is a notable overlap between the proband and the previously reported CNV case by Shono et al. [21] with the 9q34.11 deletion impacting *SET* and *SPTAN1* (without *STXBP1*). Whilst SNV/indel cases of *SET*‐NDD demonstrate mild to moderate degrees of ID, only the proband and the Shono et al. [21] deletion case exhibit severe ID (Table 1). Additionally, both the Shono et al. [21] case and Patient 2 in Stevens et al. [10] were not ‘toilet‐trained’ at the times of publication. Details were not specified in published literature, but our proband did develop severe constipation over time, requiring medical intervention by age 28. Further case reports may help to establish whether there may be a bowel component to the *SET‐*NDD phenotype in some patients as disease progresses. Regardless, our proband's deletion affects a smaller region including *SET* but not *SPTAN1* for a similar NDD phenotype to that seen by Shono et al. [21], further refining the critical haploinsufficiency interval.

In comparison, cases with 9q34.11 deletions involving *STXBP1* seem to display slightly different NDD phenotypes, often with features of epileptic encephalopathy. Notably, one such case did have a differential diagnosis of atypical RTT [48], like our patient. However, the 960 kb deletion in that report did not impact *SET*, and that patient displayed intractable epilepsy of which our proband is not affected. Further case reports of *SET*‐impacting 9q34.11 deletions may better establish the clinical link with atypical RTT, including analysis of other possible modifier variants outside of 9q34.11 which may be modulating the phenotype towards an atypical RTT picture.

## 5 Conclusion

Ultimately, this study establishes *SET* as a critical haploinsufficient gene at the 9q34.11 locus through identification of the smallest reported deletion in this region associated with NDD, not including other prominent candidate genes such as *STXBP1* and *SPTAN1*. The proband's clinical presentation expands the phenotypic spectrum of *SET*‐NDD to include features overlapping with atypical RTT and emphasises the importance of assessing and managing speech delay with early intervention over time. This study documenting the diagnostic odyssey of our patient provides valuable insights for genetic counselling and speech therapeutic interventions for affected individuals.

## Funding

This work was supported by the Victorian Government's Operational Infrastructure Support Program, MCRI Near Miss grant, Royal Children's Hospital Foundation, Australian National Health and Medical Research Council (NHMRC) Investigator Fellowships (GNT2009732), Australian Medical Research Future Fund Genomics Health Futures Mission (2016030) and Mito Foundation.

## Conflicts of Interest

The authors declare no conflicts of interest.

## Supporting information


**Table S1:1** Angelman/Rett like syndromes gene panel (v1.14) [1,2] used for the variant prioritisation of the singleton WGS data.
**Table S2:1** Synopsis of Neul's revised diagnostic criteria [3] used for the classification of typical and atypical Rett syndrome (RTT) cross‐checked with the proband.
**Table S3:1** List of HGNC‐named protein‐coding genes involved in the *de novo* deletion at chromosome 9q34.11 in the proband.
**Figure S3:1** Multiple sequence alignment showing evolutionary conservation of SET.
**Figure S4:1** Integrative Genomics Viewer (IGV) screenshot for the *NUP188* gene.
**Figure S4:2** NUP188‐targeted Western blot results.
**Figure S4:3** Scatter‐plot of NUP188 Western blot band intensities normalised to β‐actin loading control.
**Figure S4:4** NUP188 and total nucleopore complex (NPC) immunostaining in patient fibroblasts.
**Figure S4:5** No changes to total NUP188 per nucleus number or NUP188‐containing NPC density in patient fibroblasts.
**Figure S4:6** No changes in global nuclear pore density in patient fibroblasts.
**Figure S4:7** No changes in NUP188 staining intensity in nuclear area.
**Figure S4:8** No changes in nuclear area and nuclear size (based on surface area).
**Figure S4:9** Decreased level of NUP188 peptides compared to controls.

**Table S2:2** Clinical features and genomic details of previously reported neurodevelopmental disorder patients harbouring *SET* variants

## Data Availability

The data that support the findings of this study are available from the corresponding author upon reasonable request.
