# Supplementary material for: Elucidating the Role of SET as a Key Contributor to Neurodevelopmental Disability Within the 9q34.11 Deletion Syndrome Interval
Source: Clin Genet. 2026 Jul 10;110(4):438–48. doi: 10.1111/cge.70213 (PMC13533875; doi:10.1111/cge.70213)
Supplement: Supplementary file 1 [file CGE-110-438-s001.docx]

**SUPPLEMENTARY DATA:**

***S1. GENE PANEL USED FOR SINGLETON WGS***

The Angelman Rett like syndromes gene panel (v1.14) was used for variant prioritisation and curation of the singleton WGS data.

**Table S1.1: Angelman/Rett like syndromes gene panel (v1.14) [1,2] used for the variant prioritisation of the singleton WGS data.**

| **Green genes** | **Amber genes** |
| --- | --- |
| *ACTL6B, ADSL, ASXL3, ATRX, CDKL5, DYRK1A, EEF1A2, EHMT1, FOXG1, GABBR2, GRIN2B, HECTD4, HECW2, HERC2, IQSEC2, KANSL1, KCNA2, KCNQ2, KIF1A, MBD5, MECP2, MEF2C, NTNG2, SATB2, SCN2A, SHANK3, SLC9A6, SMC1A, STXBP1, SYNGAP1, SYT1, TCF4, UBE3A, UBE3C, WDR45, ZEB2* | *SLC35F1, UBTF* |

***S2. ADDITIONAL CLINICAL DETAILS***

The proband is a 28-year-old female who was the oldest of three daughters born at term after an unremarkable pregnancy apart from mild pre-eclampsia to her non-consanguineous parents. During an examination at 24 years and 4 months of age, her weight was 65kg (75^th^ percentile), height of 154 cm (5^th^-10^th^ percentile) and head circumference of 57 cm (75^th^-98^th^ percentile).

At birth, she had normal Apgar scores and there were no early concerns. She was late to roll and sit and had undergone physiotherapy at 4.5 months of age. She was noticed to have global developmental delay from around seven months of age. She sat at 14 months, crawled at 18 months and walked at 2.5 years of age. She had some words but very limited functional speech but subsequently lost those words as well. However, at 23 years of age, she went through an intense speech therapy, to which she had gained several words and her vocabulary had improved. By age 28, although speech is still limited, she can speak some phrases and uses several noises which the family can interpret with meaning. Whilst late, this is considered to be indicative of speech recovery since regression.

She exhibited slow regression of gross and fine motor skills, but did not show any phase of motor stabilisation or recovery. She had decline in physical function and developed abnormal gait. At 14 years of age she could walk 5 kms, but by age 28 she could only walk around 200 meters with less stamina and lower stability, and was observed to get fatigued very easily.

She had diminished hand function with age: she used a pincer grip in the past but recently she was found to rake. She was able to use cutlery up until 21 years of age; however she had reduced control of cutlery with age and at present she usually uses hands for feeding or is fed by family members. She had severe behavior problems involving self-harm (such as slapping her face repeatedly) when annoyed, which was treated with a number of psychoactive medications (lyrica, risperidone, frisium) with positive results. She also showed marked involuntary head writhing movements, but had no seizures. She had an impaired sleep pattern and used to wake up several times every night, and her feet used to turn blue frequently. The proband has significant constipation, and is on medication to treat her bowel symptoms. She has been diagnosed with significant ASD, and both her siblings also had mild ASD as well.

Her brain MRI showed normal pituitary gland, multiple foci of non-specific T2 hyper intensity in the white matter but no structural brain abnormalities. A single 4mm focus of FLAIR hypersensitivity within the left frontal lobe was observed which did not enhance with contrast, nor did it display diffusion restriction. She does not exhibit any cardiac lesions, and her EEG was negative.

Extensive metabolic investigations were performed on the affected individual with normal results, including: full blood count, urea and electrolytes, thyroid function tests, iron indices, acylcarnitine and amino acid profiles in blood, urine metabolic screen amino and organic acids, transferrin isoforms, active B12, urine thin layer chromatography, lysosomal enzyme analysis, and homocysteine analysis. Prolactin 2338 levels were found to be raised, but were recorded to be secondary due to risperidone therapy. In addition, raised copper (41.7) but normal caeruloplasmin was reported. Negative genetic tests included *MECP2* testing, MLPA, mitochondrial DNA testing, *CDKL5, FOXG1,* and *POLG* testing, prompting the further study by SNP microarray, WES and LRS described in this paper.

***S2.1 CLASSIFICATION OF ATYPICAL RETT SYNDROME IN PROBAND***

**Table S2.1: Synopsis of Neul’s revised diagnostic criteria [3] used for the classification of typical and atypical Rett syndrome (RTT) cross-checked with the proband.**

| **Diagnostic Criteria** | **Classic / typical RTT** | **Atypical / variant RTT** | **Proband** |
| --- | --- | --- | --- |
| **Required:** Regression followed by recovery | Yes | Yes | Yes, partially  (only speech recovery) |
| **Main criteria (4)**  1. Partial / complete loss of acquired purposeful hand skills  2. Partial / complete loss of acquired spoken language  3. Gait abnormalities  4. Stereotypic hand movements | 4/4 (all) | ≥ 2 / 4 | First diagnosis:  3/4  (no prior mention of decline in hand skills) |
|  |  |  | 2024 follow-up:  4/4  (significant decline in hand function) |
| **Supportive criteria (11)**  1. Breathing disturbances when awake  2. Bruxism when awake  3. Impaired sleep pattern  4. Abnormal muscle tone  5. Peripheral vasomotor disturbances  6. Scoliosis/kyphosis  7. Growth retardation  8. Small cold hands and feet  9. Inappropriate laughing/screaming spells  10. Diminished response to pain  11. Intense eye-pointing communication | Not required  (although often present) | ≥ 5 / 11 | First diagnosis:  9/11  (previously no scoliosis / kyphosis and no abnormal muscle tone) |
|  |  |  | 2024 follow-up: 11/11  (now noted to show kyphosis and hypotonia) |
| **Exclusion criteria (2)**  1. Brain injury secondary to peri-/postnatal trauma, neurometabolic disease, or severe infections with neurological impact  2. Grossly abnormal psychomotor development (in the first 6 months of life) | Must exclude both (2/2) | No need to exclude (0/2) | 1/2  (Late to sit and roll, underwent physiotherapy at 4.5 months of age) |
| **Final classification:** Atypical RTT (due to the presence of 1 of 2 exclusion criteria) | | | |

S3. ASSESSMENT OF THE DELETED INTERVAL AT 9q34.11

Table S3.1: List of HGNC-named protein-coding genes involved in the *de novo* deletion at chromosome 9q34.11 in the proband.

| **Gene** | **Gene name** | **LOEUF; pHaplo** | **Function** (UniProt) | **Summarised GO molecular functions** | **OMIM condition(s)** |
| --- | --- | --- | --- | --- | --- |
| *DYNC2I2* | Dynein 2 intermediate chain 2 | 1.04; N/A | Non-catalytic component of the cytoplasmic microtubule motor complex dynein-2, involved in retrograde ciliary protein trafficking and ciliogenesis [#Q96EX3] | Dynein heavy and light chain binding | **AR:** Short-rib thoracic dysplasia 11 with or without polydactyly [#615633] |
| ***SET*** | SET nuclear proto-oncogene | **0.17;  0.94** | Multifunctional, involved in apoptosis, transcription, nucleosome assembly / the inhibition of nucleosome acetylation, and histone chaperoning [#Q01105] | DNA binding; hydroxylase activity; histone binding; protein phosphatase inhibitor/regulator activity | **AD:** Intellectual development disorder 58 [#618106] |
| *PKN3* | Protein kinase N3 | 0.99;  0.45 | Catalyses the conversion of L-seryl residues into O-phospho-L-seryl [#Q6P5Z2] | Transferase activity; ATP biding; protein (serine / threonine) kinase activity; small GTPase binding | N/A |
| *ZDHHC12* | Zinc finger DHHC-type palmitoyltransferase 12 | 1.30;  0.21 | Palmitoyltransferase which catalyses the addition of palmitate onto various protein substrates [#Q96GR4] | (Protein-cysteine S-) palmitoyltransferase activity | N/A |
| *ZER1* | Zyg-11 related cell cycle regulator | **0.24;**  **0.85** | Substrate adapter subunit of an E3 ubiquitin ligase complex that may be involved in meiosis. Also involved in proteolytic fragment clearance [#Q7Z7L7] | (Biological processes) Positive regulation of proteasomal ubiquitin-dependent protein catabolic process; protein quality control | N/A |
| *TBC1D13* | TBC1 domain family member 13 | 0.95;  0.31 | Acts as a GTPase-activating protein for RAB35, potentially involved in the regulation of insulin-induced glucose transporter translocation to the plasma membrane of adipocytes [#Q9NVG8] | GTPase activator activity; small GTPase binding | N/A |
| *ENDOG* | Endonuclease G | 1.85;  0.22 | Mitochondrial endonuclease, preferentially catalyses the cleavage of double-stranded 5hmC-modified DNA [#Q14249] | DNA/RNA endonuclease (hydrolase) activity; nucleic acid / magnesium ion binding; protein homodimerisation activity | N/A |
| *SPOUT1* | SPOUT domain containing methyltransferase 1 | 0.98;  N/A | Associates centrosomes with bipolar mitotic spindle poles during metaphase; helps in chromosome alignment; may promote centrosome maturation (via anchor protein recruitment) in early mitosis; miRNA binding and regulation [#Q5T280] | Methyltransferase activity; miRNA and RNA binding | **AR:** Neurodevelopmental disorder with poor growth, seizures, and brain abnormalities [#621154] |
| *KYAT1* | Kynurenine aminotransferase 1 | 1.07;  N/A | Catalyses the irreversible transamination of the L-tryptophan metabolite L-kynurenine to form kynurenic acid (KA) in the tryptophan catabolic pathway (KA is a broad spectrum antagonist of ionotropic excitatory amino acid receptors) [#Q16773] | Transaminase activity; lyase activity; pyridoxal phosphate binding; protein homodimerisation activity | N/A |
| *LRRC8A* | Leucine rich repeat containing 8 VRAC subunit A | 0.61;  **0.80** | Essential component of the volume-regulated anion channel (VRAC), required to maintain a constant cell volume in response to extracellular or intracellular osmotic changes [#Q8IWT6] | identical protein binding; volume-sensitive anion channel activity; cyclic GMP-AMP transmembrane transporter activity | **AD:** Agammaglobulinemia 5 [#613506] (**Note:** limited disease-gene association, red-rated in PanelAPP) |
| *PHYHD1* | Phytanoyl-CoA dioxygenase domain containing 1 | 1.21;  0.33 | 2-oxoglutarate (2OG)-dependent dioxygenase, iron-dependent catalysis of 2OG into succinate and CO₂ [#Q5SRE7] | 2OG-dependent dioxygenase activity; metal ion binding | N/A |
| *DOLK* | Dolichol kinase | 0.86;  0.19 | Catalyses CTP-mediated phosphorylation of dolichol, the terminal step in *de novo* Dol-P biosynthesis (Dol-P is a lipid carrier essential for oligosaccharide and GPI anchor synthesis) [#Q9UPQ8] | (Transferase) Dolichol kinase activity | **AR:** Congenital disorder of glycosylation, type Im [#610768] |
| *NUP188* | Nucleoporin 188 | 0.54;  **0.97** | Component of the nuclear pore complex (NPC), required for trafficking of proteins across the nuclear envelope into the nucleus [#Q5SRE5] | Structural constituent of nuclear pore; (biological processes) mRNA and protein nucleocytoplasmic transport; RNA export from nucleus | **AR:** Sandestig-Stefanova syndrome [#618804] |

AR = Autosomal Recessive; AD = Autosomal Dominant. Genes with no applicable (N/A) OMIM conditions are up-to-date as of April 2026. Gene Ontology (GO) terms are summarised. The top candidate gene for the proband’s phenotype, *SET*, is highlighted in blue. gnomAD LOEUF (loss-of-function observed / expected upper bound fraction) scores are from v4.1.1; LOEUF scores closer to zero (bold font) are more intolerant of variation. pHaplo is taken from DECIPHER v11.28 (note that pHaplo is not available for all genes); higher pHaplo scores (**bold font**) indicate a greater probability of haploinsufficiency and deletion intolerance.

**Figure S3.1 Multiple sequence alignment showing evolutionary conservation of SET.**

Sequences were aligned using Clustal Omega (URL: <https://www.ebi.ac.uk/Tools/msa/clustalo/>) using the RefSeqs of *H sapiens* (NP_001116293.1), *M musculus* (NP_076360.1), *R norvegicus* (NP_001012522.1), X tropicalis (NP_989041.1), *G gallus* (NP_001025862.2), *P troglodytes* (XP_024201677.1), *S scrofa* (NP_001231019.1), and *E caballus* (XP_023484824.1).

***S4. INVESTIGATION AND EXCLUSION OF NUP188 CANDIDACY***

**PREFACE ON *NUP188***

The nucleopore complex (NPC) gene *NUP188* partially falls within the proband’s 9q34.11 deletion interval, and was initially suggested as a potential candidate gene prior to the discovery of autosomal recessive Sandestig-Stefanova syndrome (OMIM #618804) in 2020, which is a multisystemic severe neurodevelopmental (NDD) with additional craniofacial defects, ophthalmological anomalies, and congenital heart defects, which may be lethal in childhood due to respiratory failure [4,5]. *NUP188* is an inner scaffold nucleoporin which maintains NPC structural integrity, enabling nucleocytoplasmic transport and macromolecular exchange of proteins and mRNA across the nuclear membrane for downstream roles in chromatin organisation and transcriptional regulation [4,5]. As studies of our proband continued into 2021, *NUP188* was highlighted as potentially relevant (neuro-)developmental candidate gene, due to its substantial disruption by the distal breakpoint of the proband’s deletion. Whole exome sequencing (WES) also identified an additional paternally-inherited missense variant in *NUP188* [NM_015354.3:c.3922C>T, p.(Arg1308Cys)] in the proband. Hence, we aimed to implement long read sequencing (LRS) to identify the phase of the proband’s large 9q34.11 deletion with the missense variant, and perform additional functional validation to assess whether *NUP188* disruption could be contributing in some way to the NDD phenotype of the proband, acknowledging the very limited developmental phenotypic overlap of our case with the more severe and multisystemic Sandestig-Stefanova syndrome. The following molecular investigations demonstrate why *NUP188* candidacy was subsequently excluded for our proband in favour of *SET* haploinsufficiency, which eventually became a significantly better phenotypic fit for our proband as newer *SET*-NDD literature was published over time.

**METHODS**

**Cell Culture**

Primary cultures of fibroblasts from the proband and unrelated control fibroblasts in Family 1 were established from skin biopsies as previously described [6]. Fibroblasts were cultured in basal medium (high-glucose DMEM (Gibco) with 10% foetal bovine serum (Gibco), 100 units/mL penicillin, and 100 µg/mL streptomycin) at 37°C with 5% CO_2_. All fibroblast control cell lines were established in-house and were established from paediatric individuals without any suspected genetic disorders. See the main text of this publication for further details (‘*Subjects and Methods: Fibroblast Tissue Culture and Cell Harvesting*’).

**Western Blot**

SDS-PAGE (8%) Western blot analyses of NUP188 protein levels were performed in triplicate on whole cell lysates (WCL) from cultured fibroblasts derived from the proband and three independent control samples. Optimisation of Western blot conditions first discerned the appropriate WCL loading amount (15μL) and NUP188 antibody dilution levels (data not shown but available upon request). Primary antibodies used were C-terminal-targeting polyclonal anti (α)-NUP188 (Abcam: #ab86601) optimised for a dilution level of 5% skim milk powder in PBS with 0.1% Tween-20 optimised at a 1:200 ratio and overnight incubation at 4°C, with subsequent secondary α-Rabbit HRP-linked IgG (Cell Signalling Technology: #7074S) at 1:5000 for 1 hour at room temperature. The housekeeping protein β-actin (ACTB) was also probed as a loading control for each blot at a 1:10,000 dilution of monoclonal primary antibody α-ACTB (Sigma: #A1978) with secondary α-Mouse HRP-linked IgG (Invitrogen: #A16066) at 1:10,000, both for 1 hour at room temperature respectively. Samples were run alongside 20μL of 10-250kD Precision Plus Protein™ WesternC™ Standard (BioRad: #1610399), and the Hyperfilm system (Amersham: #28906837) with activated Clarity™ Western electrochemiluminescence (ECL) substrate (BioRad: #1705060). Developed films were digitally scanned, and NUP188 protein band signal intensities were normalised to ACTB loading controls within each lane using peak-areas using the FIJI-ImageJ software (<https://imagej.net/software/fiji>). Statistical analyses of unpaired t-tests comparing patient WCL mean normalised NUP188:ACTB expression to each control-line WCL was performed in the GraphPad Prism® software (<https://www.graphpad.com>), and scatter plots were generated (where errors bars represented the standard deviation of the mean (± SD as indicated).

**Immunofluorescence Microscopy**

Fibroblasts were seeded at a density of 40,000 cells per 12mm coverslip in basal medium (high-glucose DMEM with 10% foetal bovine serum, 100 units/ml penicillin and 100ug/ml streptomycin) and allowed to attach overnight at 37°C and 5% CO_2_ in a humidified incubator. Cells were washed with warm PBS and permeabilised with 0.5% Triton X-100 on ice for 5 minutes. Cells were washed with PBS again and fixed with 4% paraformaldehyde for 20 minutes at room temperature. Cells were washed twice with PBS-Tween (0.05%) before being blocked with 1% bovine serum albumin at room temperature for 1 hour. Antibodies of α-NUP188 (ThermoFisher: #A302-322A) at 1:1000 and α-MAb414 (Abcam: #ab24609) at 1:500 were diluted in blocking solution and cells were incubated at room temperature for 1 hour. Cells were washed three times for 5 minutes with PBS before being incubated in secondary antibody at room temperature for 1 hour (goat α-rabbit 488, ThermoFisher: #A-11034 1:1000 for NUP188, and goat α-mouse 594, ThermoFisher: #A-11032 for MAb414). Coverslips were mounted onto microscope slides using ProLong™ Gold Antifade Mountant with DAPI (ThermoFisher: #P36935) overnight. Images were captured using a ZEISS LSM 900 Airyscan confocal microscope and analysed using Imaris software.

Immunofluorescence (IF) images were then captured as previously described [7]. Cells were imaged with a Zeiss LSM 900 confocal microscope with Airyscan 2 super resolution imaging. NUP188 and MAb414 immunostained-cells were captured with a 63x oil immersion lens, and Z-stacks with a series of 30 slices. For NUP188 and MAb414 quantification, images were loaded in Imaris x64 (Version 9.7.1) software and an analysis pipeline was generated for batch processing. A 3D surface was created from the DAPI channel using the “*Surfaces*” function. This was used to mask the channel containing NUP188 or MAb414 staining, to exclude any instances of cytoplasmic staining. The masked channel was used for quantification of pore density using the “Spots” function. The total number of spots per nucleus was normalized to the surface area of the nucleus to calculate the pores/µm^2^. Statistical analyses were then carried out using either a two-tailed Student’s t-test or one-way ANOVA corrected for multiple comparisons as appropriate (in GraphPad Prism®). Error bars represented the standard deviation of the mean (± SEM or SD as indicated). A p-value < 0.05 was considered statistically significant.

**Mass Spectrometry-based Proteomics Analysis**

Proteomic sample processing and analysis was performed on patient fibroblasts alongside 5 fibroblast controls, as previously described and discussed in the main text of this publication (‘*Subjects and Methods: Proteomic Investigation*’) [8].

**RESULTS**

***NUP188* Missense Variant Re-Analysis**

When the *NUP188* missense variant [NM_015354.3:c.3922C>T, p.(Arg1308Cys)] first came to attention in 2021 prior to phase checking, it was rare in gnomAD v3.1.2 with only 17 heterozygotes and no homozygotes (in 152112 total alleles), restricted only to the African/African American and Admixed American ancestral datasets, different to the Caucasian ancestry of this proband. Predictive *in silico* tools of the time (SIFT, PolyPhen2, MutationTaster) classified the variant as damaging, and both the CADD v1.4 score (31) and Grantham distance (180) were high. However, upon re-analysis in 2024, this variant now has more controls in gnomAD v4.1.0, with 42 heterozygotes and no homozygotes (in 1613878 alleles), including 15 of European ancestry similar to that of our proband. Additionally, the more-modern computational tool, REVEL scored only 0.294, which is now classified as being indeterminate (neither damaging nor benign) by current ClinGen guidelines rather than the damaging suggestions from classical tool-sets [9,10]. Over time, the CADD score has also decreased according to the more recent version (27.9 in v1.6).

**Phasing of the Missense Variant and Large Deletion**

In retrospect, Nanopore LRS using the ‘read-until’ method was ineffective for adequately determining the phase of the *NUP188* missense variant with the large 9q34.11 deletion in this proband, due to the small number of >15kb reads genes able to be generated. As shown in Supplementary Figure S4.1, there was only one Nanopore sequence read which was able to (and indeed did) show both the missense variant and the large deletion *in cis* (on the same allele). This is considered to be sufficient (albeit weak) support for the paternally-inherited *NUP188* variant occurring *in cis* with the larger *de novo* deletion.

**Supplementary Figure S4.1:** Integrative Genomics Viewer (IGV) screenshot for the *NUP188* gene. The breakpoint of the proband’s 9q34.11 deletion is supported by a significant change of sequence coverage (red arrow). Several reads carrying the upstream *DYNC2I2* gene sequence are mapped to the *NUP188* gene (orange arrows). The one Nanopore sequence read (pink star) is shown which carries both the missense variant in the *NUP188* gene (pink arrows) and the deletion genomic breakpoint. Mismatch sequences that are not mapped to the *DYNC2I2* gene are pointed by dark grey arrows.

**Western Blot results**

Western blot functional validation in triplicate showed a statistically significant decrease in NUP188 protein levels in the proband compared to independent control fibroblasts, but not a complete loss of NUP188. There appears to be residual expression of the NUP188 protein as demonstrated by the faint band present (Supplementary Figure S4.2; ‘Patient’ lanes), further supporting only a partial depletion of NUP188 (which is consistent with the heterozygous deletion). As shown in Supplementary Figure S4.3, separate unpaired t-tests comparing patient mean normalised NUP188:ACTB expression to each control demonstrate this is a variably statistically significant decreased expression level compared to controls, but still show *some* expression of NUP188 (as the patient values are not completely zero, which might be expected for complete loss of NUP188).

**Supplementary Figure S4.2:** NUP188-targeted Western blot results. ECL-developed films for Western blots performed for three independently extracted sets of WCLs from fibroblasts (n=3). Each set contains 15μg of WCLs from the independent control lines (control 1, 2, and 3) and proband skin fibroblasts (‘Patient’). Top panels show blots probed with a 1:200 dilution of primary α-NUP188 antibodies (Abcam: #ab86601; labelled at expected 188kD size) at 4°C overnight, followed by secondary α-Rabbit antibodies (Cell Signalling Technology: #7074S) at a 1:5000 dilution for 1 hour at room temperature. Bottom panels show β-actin loading control probing using 1:10,000 dilutions of both primary α-ACTB (Sigma-Aldrich: #A1978; labelled at expected 42kD size) and secondary α-Mouse (Invitrogen: #A16066) antibodies for 1 hour at room temperature. Approximated molecular weights are shown on the right from the 10-250kD Precision Plus Protein™ WesternC™ Standard (BioRad: #1610399). 50kD is indicated twice due to a layout crossover between NUP188 and β-actin probing regions of interest. Exposure times for each blot are: Set 1 Fibs: α-NUP188: 6 minutes 15 seconds; α-ACTB: 7 seconds. Set 2 and 3 Fibs: α-NUP188: 1 minute 10 seconds; α-ACTB: 30 seconds. Uncropped images are available upon request.

**Supplementary Figure S4.3**: Scatter-plot of NUP188 Western blot band intensities normalised to β-actin loading control. ‘Normalised NUP188:β-actin’ band intensities (normalised intensity arbitrary units) are shown for each control (control 1, 2, and 3) and patient WCL extract. Each point represents the normalised intensity for that WCL extract from one of three replicate Western blots (independent WCL Sets 1/2/3), and are shown in a vertical line unless doing so would obstruct another data point, in which case they were offset in the X-direction (Control 800169, Patient). Error bars show mean normalised NUP188:β-actin expression (central horizontal line) ± standard deviation (top/bottom horizontal lines) within each sample. Statistical analyses shown are for separate unpaired t-tests comparing patient mean normalised NUP188:β-actin to those of each control, with p-values (‘p-val’) for each test shown. Significance p-value thresholds were: * = p-value < 0.05; ** = p-value < 0.01; *** = p-value < 0.001. Original intensities were measured and normalised using FIJI (ImageJ). Scatter-plot made using GraphPad Prism®.

**Immunofluorescence results**

Immunofluorescence (IF) imaging indicated similarities between proband cells and multiple independent controls (Supplementary Figure S4.4). No statistically significant changes in total global nuclear pore density were found (where the NUP188 protein is localised, functioning in nucleocytoplasmic transport of mRNA and proteins) for our proband’s fibroblast cells (Supplementary Figure S4.5 & S4.6). There were also no statistically significant changes in nuclear area or size (Supplementary Figure S4.7 & S4.8) (if this protein were impaired, one would expect a swelling in nuclear size due to the impaired, blocked nucleocytoplasmic transport – however, in our proband’s cells, this does not appear to be the case). Lastly, there were no changes in NUP188 staining intensity in the nuclear area, suggesting this protein is still expressed where it is functionally required to a sufficient enough degree. These IF results suggest no significant impact of the proband’s *NUP188* variants on functional NUP188 activity at the protein level.

**Supplementary Figure S4.4**: NUP188 and total nucleopore complex (NPC) immunostaining in patient fibroblasts. Compressed Z-stack representative immunostained images of NUP188 (green spots), MAb414 (red spots) and for comparison nucleus (DAPI; blue spots) in patient fibroblasts and controls (BLCcont1, 2, 4, 5, and 7). The merge of all three is shown in the final column (light blue).

**Supplementary Figure S4.5**: **No changes to total NUP188 per nucleus number or NUP188-containing NPC density in patient fibroblasts:** Quantification of **(A)** NUP188 number per nucleus and **(B)** NUP-containing NPC pore density (pores per µm^2^ of nuclear surface area) in patient fibroblasts compared to controls shows no consistent difference in patient fibroblasts. N=1 biological samples/cell line, pooled data from >24 nuclei/cell line. One-way ANOVA with Sidak’s multiple comparisons test; ****p<0.001*, mean ± SEM.

**Supplementary Figure S4.6**: **No changes in global nuclear pore density in patient fibroblasts:** Quantification of **(A)** MAb414 number per nucleus and **(B)** MAb414 immunostained NPC pore density (pores per µm^2^ of nuclear surface area) in patient fibroblasts compared to controls shows no consistent changes in NPC pore density in patient fibroblasts compared to three of the four controls. N=3 biological samples/cell line, pooled data from >80 nuclei/cell line. One-way ANOVA with Sidak’s multiple comparisons test; ** *p<0.01*, ****p<0.001*, mean ± SEM.

**Supplementary Figure S4.7**: **No changes in NUP188 staining intensity in nuclear area:** Quantification of staining intensity with **(A)** mean staining intensity and **(B)** integrated intensity in patient fibroblasts compared to controls shows no consistent changes in patient fibroblasts compared to three of the five controls. N=3 biological samples/cell line, pooled data from >80 nuclei/cell line. One-way ANOVA with Sidak’s multiple comparisons test; ** *p<0.01*, ****p<0.001*, mean ± SEM.

**Supplementary Figure S4.8**: **No changes in nuclear area and nuclear size (based on surface area):** Quantification of nuclear surface area (µm^2^) in patient fibroblasts compared to controls shows no consistent changes in patient fibroblasts compared to four of the five controls. N=3 biological samples/cell line, pooled data from >80 nuclei/cell line. One-way ANOVA with Sidak’s multiple comparisons test; ** *p<0.01*, ****p<0.001*, mean ± SEM.

**Mass Spectrometry Proteomics Results**

Overall, peptide levels across the proband’s deleted 9q34.11 interval are significantly decreased (Supplementary Figure S3). The relative abundance of NUP188 peptides from the patient-derived fibroblasts are slightly decreased compared to the control lines (n=5) on the MS2 intensity plot (Supplementary Figure S4.9A). The number of (trypsin-cleaved) peptides containing the canonical p.(Arg1308) peptide is decreased to similar levels as other NUP188 peptides (Supplementary Figure S4.9B), suggesting the reduced levels of NUP188 are consistent across the entire protein and do not seem to be affected by the missense variant (Supplementary Figure S3B). The disparity between the western blotting and proteomics analysis can be attributed the higher sensitivity of the proteomics analysis.

**Supplementary Figure S4.9**: **Decreased level of NUP188 peptides compared to controls.** Panel **(A)** shows MS2 quantities (z-scored intensities) for NUP188 across all quantitative proteomic samples. Raw MS2 intensity levels of identified proteins from quantitative proteomic data analysed in the same sample batch were log_2_ transformed. Relative levels of NUP188 protein within each sample were highlighted in red (line). **(B)** Peptide levels (red line; difference log2 MS2 quantity NUP188 peptides) across the length of the NUP188 protein sequence (X-axis indicating amino acid position), for proband vs controls (N = 5) using loess method with 95% confidence interval. The peptide containing the p.Arg1308 residue (location shown by arrow) is detected in the patient at reduced levels. For both figures, N patient replicates = 3 and N controls = 5 as shown.

**DISCUSSION**

Ultimately, the multiple lines of functional validation experiments focussed on NUP188 demonstrate that although NUP188 protein levels were reduced in proband fibroblasts on both Western blots and proteomic investigation, there still appears to be sufficient residual expression, which is concordant with the immunofluorescence data showing no alterations to NUP188 localisation and density, nor to nuclear area and size. This would suggest that even though there is a decrease in NUP188 levels as expected as a consequence of one copy of the gene being partially deleted (which is also the allele containing the p.(Arg1308Cys) missense variant *in cis*), there is still sufficient expression from the wildtype allele in this heterozygous proband for sufficient NUP188 protein to be generated to fulfil its function. As such, *NUP188* as a candidate gene for the proband’s neurodevelopmental phenotype was excluded based on these functional studies, in favour of other NDD-associated genes within the 9q34.11 interval which were deleted (specifically *SET*, as discussed in the main publication).

This decrease in suspicion for *NUP188* as the candidate disease gene is not clinically unreasonable, given existing recessive NDD conditions associated with the *NUP188* gene do not overlap greatly with the proband’s phenotype. Although the severe level of ID is a clinical match, other variable severe multisystemic features are absent in our proband. For instance, those impacted by Sandestig-Stefanova syndrome (OMIM #618804) may display congenital heart defects, such as patent ductus arteriosus (PDA), ventricular septal defects, bicuspid aortic valves, and other cardiovascular abnormalities [4,5]. Our proband had no heart defects. Some Sandestig-Stefanova syndrome cases may also show ophthalmological anomalies (for instance microphthalmia, optic atrophy, cataracts, etc.) as well as characteristic striking facial dysmorphism [4,5], but our proband is only known to have a minor squint (strabismus) with generalised facial asymmetry and some facial dysmorphism (see main publication for clinical pictures). Other key symptoms of Sandestig-Stefanova syndrome are not a clinical match with our proband, including hearing loss, seizures, clefting / palatal abnormalities, and respiratory failure which is often associated with early lethality [4,5]; our proband is now in her late 20s and does not display any such anomalies. Indeed, our proband was in any case not found to be bi-allelic for *NUP188* loss-of-function variants, which is required for the recessive mode of inheritance of Sandestig-Stefanova syndrome, as the large deletion and missense were found to be *in cis* as shown by LRS data. However, it should be acknowledged the significant limitation of the ‘read-until’ LRS method as implemented here was unable to cover the region required for a high level of certainty. Regardless, although the monoallelic state of *NUP188* deficiency in this proband might hypothetically confer a ‘milder phenotype’ in this proband, our functional studies do not support significant dysfunction of the NUP188 protein and so do not support this hypothesis. Further studies might uncover a modifier effect of decreased NUP188 abundance in modulating the predominantly *SET*-conferred phenotype; protein-protein interaction (PPI) studies to identify the links (if any) between NUP188 and SET biological pathways may be a possible avenue of future investigation to discern if this is a reasonable suggestion.

Overall, we conclude that the likelihood of *NUP188* contributing to the NDD phenotype of our proband is very unlikely, with *SET* haploinsufficiency being a much better clinical match, for which there are documented cases of autosomal dominant NDD in the literature, including for 9q34.11 deletions of similar intervals (see main publication).

**Supplementary References**
1. Martin AR, Williams E, Foulger RE, Leigh S, Daugherty LC, Niblock O, et al. PanelApp crowdsources expert knowledge to establish consensus diagnostic gene panels. Nat Genet. 2019;51:1560–5. https://doi.org/10.1038/s41588-019-0528-2

2. Robertson AJ, Tran K, Patel C, Sullivan C, Stark Z, Waddell N. Evolution of virtual gene panels over time and implications for genomic data re-analysis. Genet Med Open. 2023;1:100820. https://doi.org/10.1016/j.gimo.2023.100820

3. Neul JL, Kaufmann WE, Glaze DG, Christodoulou J, Clarke AJ, Bahi-Buisson N, et al. Rett Syndrome: Revised Diagnostic Criteria and Nomenclature. Ann Neurol. 2010;68:944–50. https://doi.org/10.1002/ana.22124

4. Sandestig A, Engström K, Pepler A, Danielsson I, Odelberg-Johnsson P, Biskup S, et al. NUP188 Biallelic Loss of Function May Underlie a New Syndrome: Nucleoporin 188 Insufficiency Syndrome? Mol Syndromol. 2020;10:313–9. https://doi.org/10.1159/000504818

5. Muir AM, Cohen JL, Sheppard SE, Guttipatti P, Lo TY, Weed N, et al. Bi-allelic Loss-of-Function Variants in NUP188 Cause a Recognizable Syndrome Characterized by Neurologic, Ocular, and Cardiac Abnormalities. Am J Hum Genet. 2020;106:623–31. https://doi.org/10.1016/j.ajhg.2020.03.009

6. Fowler KJ. Storage of skin biopsies at -70 degrees C for future fibroblast culture. J Clin Pathol. 1984;37:1191–3. https://doi.org/10.1136/jcp.37.10.1191

7. Van Bergen NJ, Bell KM, Carey K, Gear R, Massey S, Murrell EK, et al. Pathogenic variants in nucleoporin TPR (translocated promoter region, nuclear basket protein) cause severe intellectual disability in humans. Hum Mol Genet. 2022;31:362–75. https://doi.org/10.1093/hmg/ddab248

8. Hock DH, Caruana NJ, Semcesen LN, Lake NJ, Formosa LE, Amarasekera SSC, et al. Untargeted proteomics enables ultra-rapid variant prioritisation in mitochondrial and other rare diseases. Genome Med. 2025;17:58. https://doi.org/10.1186/s13073-025-01467-z

9. Richards S, Aziz N, Bale S, Bick D, Das S, Gastier-Foster J, et al. Standards and Guidelines for the Interpretation of Sequence Variants: A Joint Consensus Recommendation of the American College of Medical Genetics and Genomics and the Association for Molecular Pathology. Genet Med. 2015;17:405–24. https://doi.org/10.1038/gim.2015.30

10. Pejaver V, Byrne AB, Feng B-J, Pagel KA, Mooney SD, Karchin R, et al. Calibration of computational tools for missense variant pathogenicity classification and ClinGen recommendations for PP3/BP4 criteria. Am J Hum Genet. 2022;109:2163–77. https://doi.org/10.1016/j.ajhg.2022.10.013
